# *Helicobacter pylori*-targeted AI-driven vaccines: a paradigm shift in gastric cancer prevention

**DOI:** 10.3389/fimmu.2024.1500921

**Published:** 2024-11-28

**Authors:** Zhiwei Tu, Youtao Wang, Junze Liang, Jinping Liu

**Affiliations:** State Key Laboratory of Oncology in South China, Guangdong Provincial Clinical Research Center for Cancer, Sun Yat-sen University Cancer Center, Guangzhou, China

**Keywords:** gastric cancer, *Helicobacter pylori*, precision medicine, targeted therapy, neoantigen vaccines, artificial intelligence

## Abstract

*Helicobacter pylori (H. pylori)*, a globally prevalent pathogen Group I carcinogen, presents a formidable challenge in gastric cancer prevention due to its increasing antimicrobial resistance and strain diversity. This comprehensive review critically analyzes the limitations of conventional antibiotic-based therapies and explores cutting-edge approaches to combat *H. pylori* infections and associated gastric carcinogenesis. We emphasize the pressing need for innovative therapeutic strategies, with a particular focus on precision medicine and tailored vaccine development. Despite promising advancements in enhancing host immunity, current *Helicobacter pylori* vaccine clinical trials have yet to achieve long-term efficacy or gain approval regulatory approval. We propose a paradigm-shifting approach leveraging artificial intelligence (AI) to design precision-targeted, multiepitope vaccines tailored to multiple *H. pylori* subtypes. This AI-driven strategy has the potential to revolutionize antigen selection and optimize vaccine efficacy, addressing the critical need for personalized interventions in *H. pylori* eradication efforts. By leveraging AI in vaccine design, we propose a revolutionary approach to precision therapy that could significantly reduce *H. pylori* -associated gastric cancer burden.

## 1 Introduction

*Helicobacter pylori (H. pylori)* is a ubiquitous human pathogen that colonizes the gastric mucosa of approximately half the world’s population (1). This gram-negative bacterium has been classified as a Group I carcinogen by the World Health Organization due to its pivotal role in the etiology of various gastric disorders, including chronic gastritis, peptic ulcers, and gastric adenocarcinoma (2, 3). The pathogenesis of *H. pylori*-associated Gastric cancer (GC) involves a complex interplay between microbial virulence factors, host genetic mutations and immune responses, and other exposure factors, such as high-nitrite diets, culminating in cascade of inflammatory events that can lead to severe gastric pathology.

Upon infection, *H. pylori* trigger a robust proinflammatory response, resulting in chronic gastritis. Persistent inflammation can induce gastric epithelial cell apoptosis and mucosal atrophy, setting the stage for compensatory hyperplasia and dysregulated cell differentiation. This altered cellular landscape often progresses to intestinal metaplasia, a precursor lesion that may further evolve into dysplasia, carcinoma *in situ*, and ultimately invasive adenocarcinoma (4–6). Leveraging cutting-edge Artificial intelligence (AI) tools to decipher *H. pylori*’s antigenic landscape is crucial for developing next-generation precision vaccines to combat *H. pylori*-associated malignancies.

## 2 *H. pylori:* unravling the molecular arsenal of a gastric pathogen

### 2.1 Decoding *H. pylori’s* molecular arsenal: implications for precision medicine and targeted therapy in gastric cancer

*H. pylori*’s pathogenicity is primarily driven by two key virulence factors: the vacuolating cytotoxin A (VacA) and the *cag* pathogenicity island (cagPAI)-encoded type IV secretion system (T4SS), along with its effector protein CagA (2, 7). Upon infection, these factors orchestrate a complex immune response characterized by a mixed Th1/Th17-mediated pro-inflammatory cascade. The T4SS facilitates the translocation of CagA into gastric epithelial cells (8–10) triggering chronic inflammation and activating pro-inflammatory signaling pathways, including nuclear factor-κB (NF-κB) and signal transducer and activator of transcription 3 (STAT3) (11). Concurrently, VacA activates the epidermal growth factor receptor (EGFR), initiating AKT and WNT/β-catenin signaling cascades that promote cell proliferation and oncogenic transformation (12, 13). These molecular mechanisms collectively drive *H. pylori*-induced gastritis and gastric carcinogenesis, which have emerged as key focal points in the landscape of precision medicine and targeted therapy.

Recent studies have unveiled the profound impact of *H. pylori* infection on the gastrointestinal microbiome. Infected individuals exhibit a distinct dysbiosis characterized by an enrichment of *Proteobacteria*, particularly within the *Epsilonproteobacteria* class, *Campylobacterales* order and *Helicobacteraceae* family, and *Helicobacter* genus. This alteration in microbial community structure is closely associated with *H. pylori* colonization (14–16) and its ability to modulate pH through urease activity. The resulting neutral microenvironment facilitates further microbial imbalances, potentially contributing to carcinogenesis.

Intriguingly, the oncogenic *H. pylori* extend beyond gastric malignancies. Emerging evidence suggests associations with colorectal, pancreatic, and hepatobiliary cancers (17–21), underscoring the far-reaching implications of this pathogen in gastrointestinal oncology. These findings highlight the need for a comprehensive understanding of *H. pylori*’s systemic effects and emphasize the importance of effective eradication strategies, including targeted therapy, in cancer prevention.

## 3 Global gastric cancer trends: mapping the *H. pylori* connection

Gastric Cancer represents a significant global health burden, ranking as the fifth most common cancer worldwide and a leading cause of cancer-related mortality. Analysis of data from The Global Cancer Observatory database (https://gco.iarc.fr/en/projects) reveals striking geographical variations in the age-standardized incidence rate (ASIR) of GC, with particularly high rates observed in Asia and neighboring regions. Notably, countries such as Japan (27.6/100,000), Azerbaijan (16.56/100,000), and China (13.72/100,000) exhibit elevated ASIRs, which are strongly associated with *H. pylori* infection and dietary factors.

**Figure 1:**
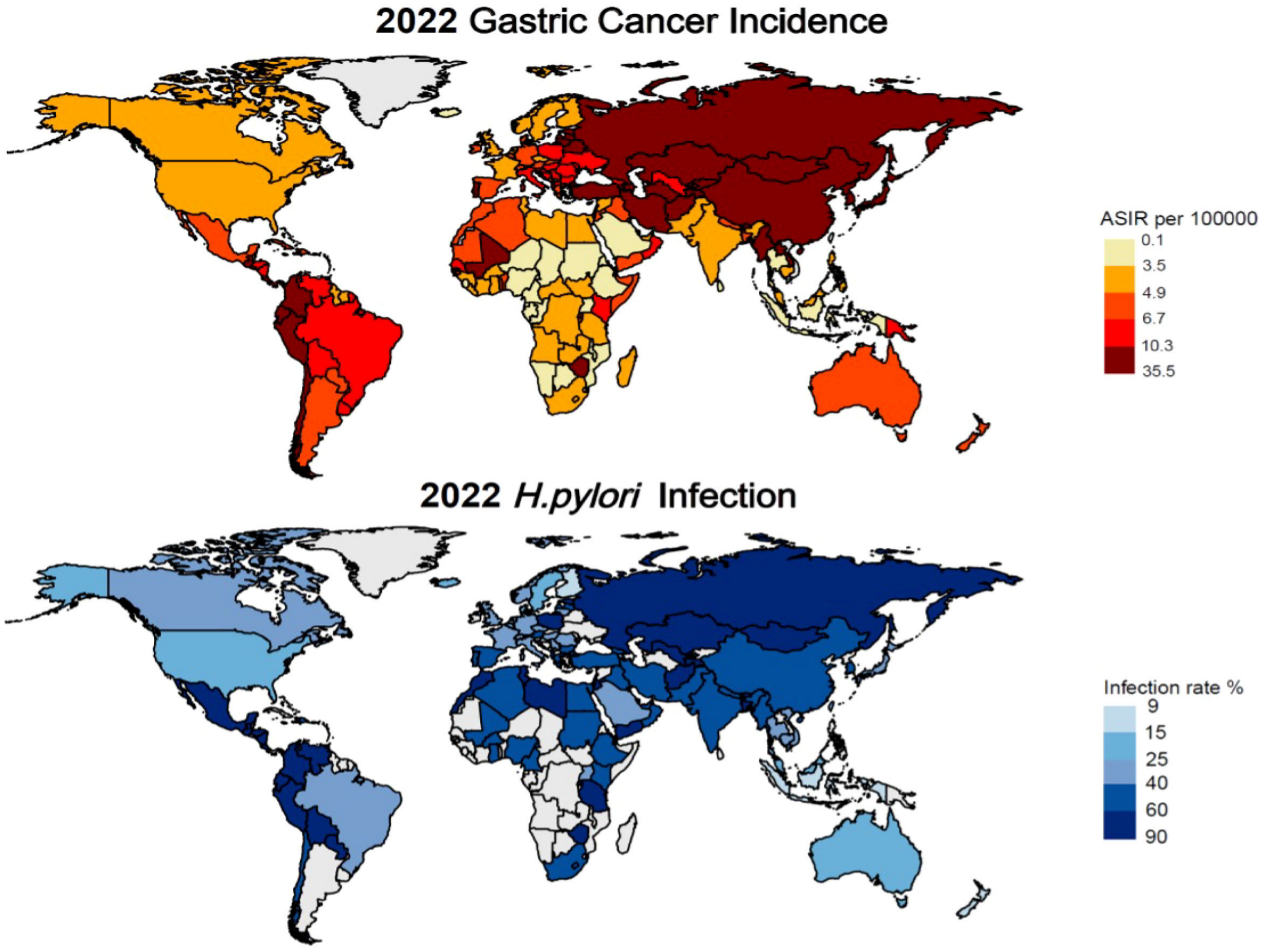
Latest global prevalence of gastric cancer incidence and *Helicobacter pylori* infection (as of 2022).

The etiology of GC is multifactorial, with *H. pylori* infection identified as primary risk factor, alongside alcohol consumption and smoking. Dietary habits, particularly consumption of high-nitrite salt-preserved foods and low-fruit intake (22), like South Korea (26.98/100,000) and North Korea (13.91/100,000). In contrast, regions with lower *H. pylori* infection rates, such as North America and Europe, demonstrate comparatively lower GC incidence, exemplified by Finland (3.99/100,000) and Canada (4.65/100,000) (**Figure 1**).

A comprehensive study examining *H. pylori* infection prevalence and gastric cancer incidence from 1980 to 2022 revealed significant regional variations. The highest infection rates among adults are observed in Africa and the Eastern Mediterranean, while lower rates were reported in Asia and Europe. Countries such as Jordan (88.6%), Ecuador (85.7%), and Guatemala (86.6%) exhibit the highest prevalence of infection. Conversely, countries like Finland (9.1%), and New Zealand (15.0%) demonstrate markedly lower infection rates (23, 24).

The globally prevalence of *H. pylori* and its role in initiating inflammatory responses that can lead to carcinogenesis underscore the critical need for effective management strategies. This review provides a comprehensive overview of current treatment approaches and explores innovative therapeutic strategies aimed at mitigating *H. pylori* infection and its associated health risk.

## 4 Rethinking *H. pylori* eradication: beyond antibiotics

Eradication of *H. pylori* remain the cornerstone of gastric disease prevention. Current treatment strategies primarily rely on empirical antibiotic regimens, with triple therapy as the gold standard. Despite achieving high eradication rates (>90%) in some populations (25), the global surge in antibiotic resistance poses a significant challenge. Triple therapy is no longer considered the gold standard in many regions (26). This escalating threat has spurred the development of more robust approaches, including quadruple therapy and adjunctive treatments, to combat this resilient pathogen.

### 4.1 Triple and quadruple therapies

*H. pylori* eradication strategies have evolved significantly since the introduction of proton pump inhibitor (PPI)-based triple therapy in 1997. The regimen, combining a PPI with amoxicillin and either clarithromycin, metronidazole, or levofloxacin, has been the global standard for over two decades (27). However, its efficacy has waned due to increasing antibiotic resistance particularly in China where resistance rates to metronidazole, clarithromycin, and levofloxacin have reached 78.2%, 22.1%,19.2%, respectively (28).

In response to this challenge, bismuth (subcitrate/subsalicylate)-based quadruple therapy (BQT) has emerged as a potent alternative. Comprises a PPI, bismuth, tetracycline HCl, and metronidazole, BQT achieves eradication rate between 90% and 95% when administered for 14 days (29). Recent guidelines now recommend BQT as first-line treatment in regions with high clarithromycin resistance (30, 31). Notably, a pediatric study reported a 95% eradication rate with excellent compliance using a 10-day BQT regimen (32). Despite its efficacy, BQT is not without limitations. Approximately 50% of patients experience side effects such as nausea and abdominal pain (29). Moreover, non-bismuth quadruple therapies, while effective, risk unnecessary antibiotic exposure. The optimal choice of eradication therapy should be guided by regional antibiotic susceptibility patterns, accessibility, and cost-effectiveness considerations.

Non-bismuth quadruple therapy, encompassing concomitant and sequential regimens, combines PPI, amoxicillin, metronidazole (or tinidazole), and clarithromycin, administered over a 14-day course (33). However, this approach faces challenges in regions with high antibiotic resistance. For instance, in Houston, clarithromycin and metronidazole resistance rate of 15% and 25%, respectively, render traditional triple therapies ineffective (34). Moreover, the potential for unnecessary antibiotic exposure raises concerns about antimicrobial stewardship, leading to recommendations against its use as a primary first-line treatment (35, 36). Probiotics have emerged as a promising adjunct in the treatment of *H. pylori* infections (37, 38), offering potential benefits in modulating the gut microbiome and enhancing host immune responses (39). However, the precise mechanisms underlying the role of probiotics in preventing *H. pylori* infection remain incompletely understood. Recent studies have shed light on potential mechanisms, suggesting that probiotics can stimulate the release of inflammatory cytokines, activate the NF-κB pathway, and induce the production of IL-8 (40). Additionally, certain probiotic strains, particularly lactic acid bacteria, have been shown to inhibit *H. pylori* colonization through the synthesis of bacteriocin-related antimicrobial substances and secreting of inhibitory compounds, including organic acids, hydrogen peroxide, and carbon dioxide. Despite these promising findings, it is important to note that the role of probiotics in *H. pylori* eradication is primarily adjunctive, enhancing the efficacy of antibiotic therapy rather than serving as a standalone treatment (41, 42). This limitation underscores the critical need for novel preventive strategies, particularly in light of the growing concern surrounding antibiotic resistance. Future research should focus on elucidating the complex interactions between probiotics, *H. pylori*, and the host microbiome, with the goal of developing more effective and targeted approaches to prevent and treat *H. pylori* infections.

### 4.2 Regional Variations in *H. pylori* Antibiotic Resistance: Insights from a Comprehensive Chinese Study

A large-scale study investigating the prevalence of *H. pylori* infection and antibiotic resistance patterns across China has revealed significant regional disparities, providing crucial insights for tailoring treatment strategies (43). This comprehensive analysis, encompassing 52 municipalities across 26 provinces and involving 12,902 participants, utilized quantitative polymerase chain reaction (qPCR) to assess resistance to two key antibiotics: clarithromycin and levofloxacin.

The study uncovered an overall *H. pylori* infection rate of 27.08% among the Chinese population, with alarming resistance rates of 50.83% for clarithromycin and 47.17% for levofloxacin. Notably, antibiotic resistance patterns exhibited significant variations across demographic factors and geographical regions:

- Gender differences: Women showed higher resistance rates (53.85% for clarithromycin, 49.01% for levofloxacin) compared to men (45.48% and 43.90%, respectively), possibly due to more frequent antibiotic use in treating gynecological infections.
- Age-related trends: Middle-aged and older individuals demonstrated elevated resistance rates (54.58% for clarithromycin, 54.54% for levofloxacin), surpassing the overall resistance rate. This trend may be attributed to increased cumulative antibiotic exposure over time.
- Geographical disparities: Northern provinces, such as Heilongjiang (77.08%) and Jilin (77.91%), exhibited markedly higher resistance rates compared to southern provinces like Hunan (27.78%). These regional variations likely reflect differences in socioeconomic conditions, sanitation practices, and antibiotic usage patterns across China.

These findings underscore the critical importance of considering regional and demographic factors when developing targeted strategies for *H. pylori* eradication. The observed disparities in antibiotic resistance highlight the need for tailored treatment approaches and emphasize the urgency of implementing more stringent antibiotic stewardship programs, particularly in high-resistance regions. Future research should focus on elucidating the underlying causes of these regional variations and developing innovative strategies to combat the growing challenge of antibiotic resistance in *H. pylori* management (43).

## 5 Challenges in eradicating *H. pylori*: rethinking gastric cancer prevention

Antibiotic resistance poses a formidable challenge to *H. pylori* eradication and gastric cancer prevention. The resistance primarily stems from point mutations in antimicrobial-associated genes (e.g., rrn23S, pbp-1, rdxA.) and the presence of drug efflux pumps. For instance, mutations in the 23S rRNA can reduce clarithromycin binding affinity, while efflux pumps actively expel antibiotics, maintaining suboptimal intracellular drug concentrations (44, 45). Additionally, *H. pylori* eradication efficacy is intricately linked to colonization density and virulence factors like cagA and vacA. This pathogen induces gastrointestinal microbiota dysbiosis, altering the host’s susceptibility (46, 47). Notably, Epstein-Barr virus (EB Virus) and *H. pylori* may synergistically exacerbate gastric inflammation, potentially accelerating carcinogenesis (48). These findings underscore the complex interplay between pathogen, host, and microbiome in *H. pylori*-associated gastric pathology, highlighting the need for multifaceted therapeutic approaches.

**Figure 2:**
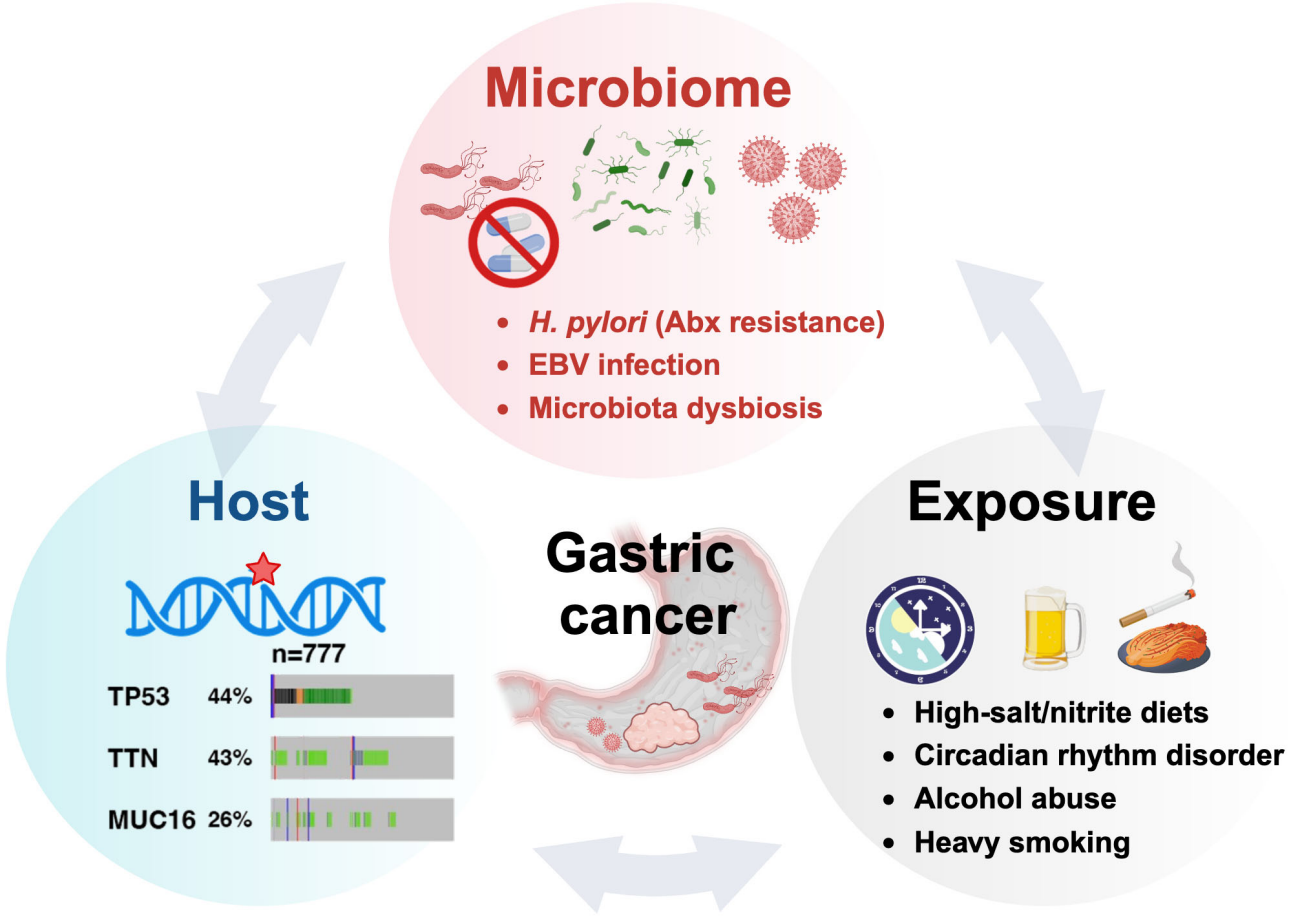
The oncogenic H. pylori interplays with the host and exposure factors in gastric carcinogenesis.

Genetic predisposition plays a crucial role in GC susceptibility. Mutations in the *CDH1* and *BRCA1*/*BRCA2* genes significantly increase risk (49). High mutation rates in *TP53* (~43%), *TTN* (~42.5%), and *MUC16* (~25.2%) underscore their potential as key drivers of GC (50–52). Additionally, lifestyle factors, such as smoking, alcohol consumption, high-salt/nitrite diets can impede *H. pylori* eradication and promote GC development (53–55) (**Figure 2**).

The multifaceted nature of *H. pylori* infection and its associated cancer risk necessitates innovative therapeutic approaches. Current strategies often fall short of complete eradication, highlighting the urgent need for novel interventions. One promising avenue involves harnessing the host’s adaptive immune system to generate specific, long-lasting memory B and T cells, potentially leading to more efficient *H. pylori* elimination.

## 6 Molecular camouflage: how *H. pylori* evades immunity in gastric cancer

*H. pylori* infection triggers a sophisticated immune response, orchestrating a complex interplay between innate and adaptive immunity. The innate immune system, acting as the first line of defense, employs pattern recognition receptors (PRRs) on gastric epithelial cells to detect pathogen-associated molecular patterns (PAMPs) (56, 57). This recognition cascade activates NOD-like receptor (NLR) and Toll-like receptors (TLRs), initiating NF-κB signaling and promoting the release of pro-inflammatory cytokines, particularly IL-8 (58).

The adaptive immune response further refines this defense strategy. Type 2 innate lymphocytes (ILC2) secrete IL-5, stimulating B cells to produce IgA, IgG, IgM antibodies. Concurrently, the *H. pylori* virulence factor CagA induces dendritic cells maturation, facilitating antigens presentation to naïve T cells and their subsequent differentiation into Th1/Th17/Tfh subsets (59).

**Figure 3:**
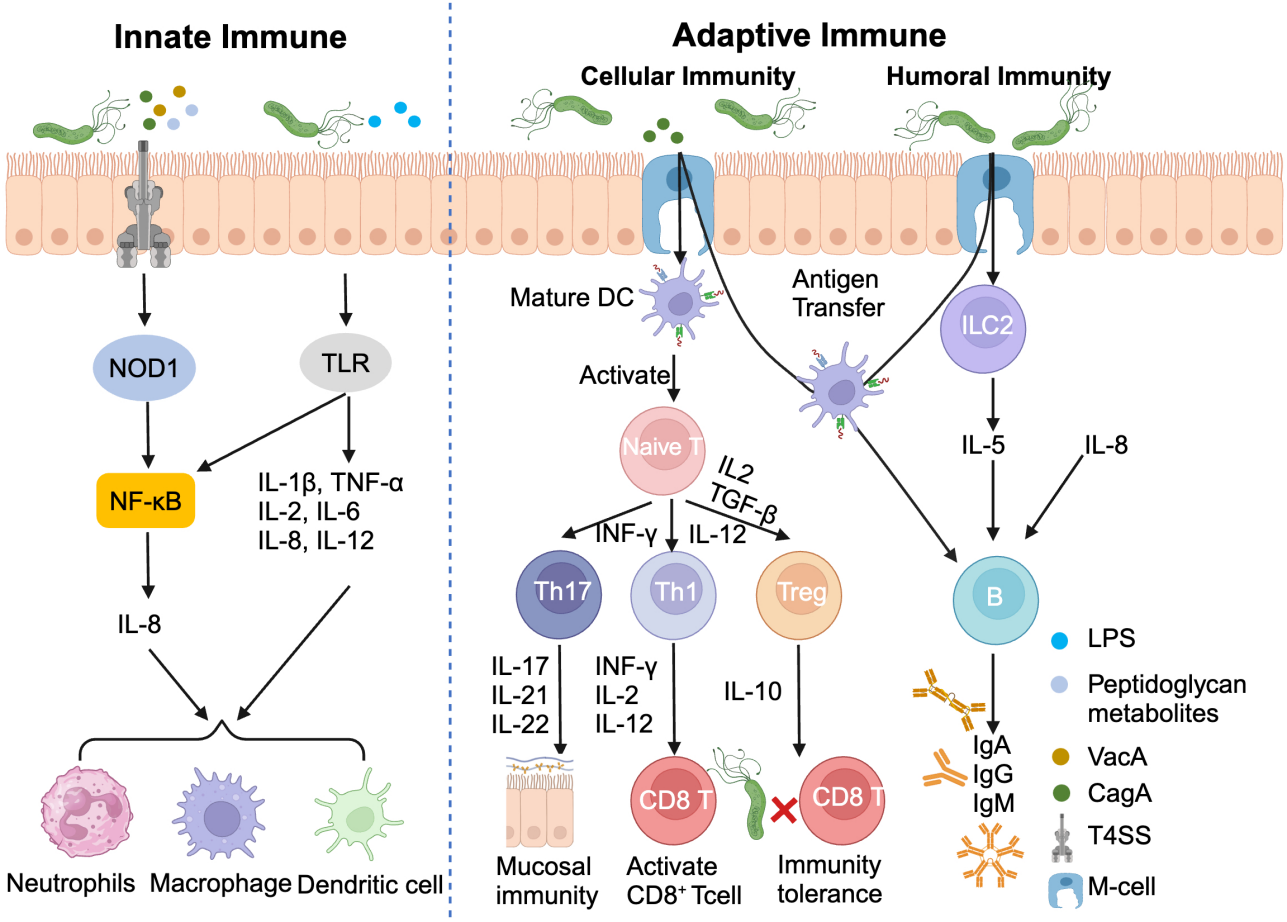
*Helicobacter pylori* infection induces innate and adaptive immunity. DC, Dendritic cell; ILC2, Type 2 innate lymphocytes; LPS, Lipopolysaccharide.

Paradoxically, *H. pylori* has evolved mechanisms to subvert this immune assault. By promoting the differentiation of Th17 cells toward regulatory T cells (Tregs), which secrete the anti-inflammatory cytokine IL-10, the bacterium ingeniously establishes immune tolerance, enabling its long-term persistence in the gastric niches (**Figure 3**) (60). This delicate balance between immune activation and evasion underscores the complexity of *H. pylori* pathogenesis and highlights potential targets for therapeutic intervention.

## 7 Harnessing host immunity in *H. pylori* vaccine development

The persistence of *H. pylori* infections, despite initial antibiotic success, underscores a critical challenge in global health. Antibiotic resistance and infection recurrence, particularly in resource-limited settings necessitate innovative strategies for sustainable eradication. Drawing parallels with the transformative impact of Human Papillomavirus (HPV) vaccines on cervical cancer prevention, the development of *H. pylori* vaccines emerges as a promising frontier in gastric cancer prevention (61, 62).

However, the genetic diversity of *H. pylori* strains presents a formidable obstacle to vaccine development. This variability, reminiscent of HPV’s subtype complexity, demands a nuanced approach to antigen selection and vaccine design (**Table 1**). In this context, precision medicine offers valuable insights by identifying strain-specific targets and tailoring immunological strategies accordingly.

**Table 1:** Partial *H. pylori* strains and their pathogenicity on human or mouse.

*H. pylori* strain	Pathogenicity	Infectivity	Molecular characteristic
SS1 (63)	Inflammation/Intestinal Metaplasia	Human/Mouse	cagA+ and vacA+
PMSS1 (63)	Inflammation/Metaplasia	Human/Mouse	cagA+ and vacA+
ATCC49503 (63)	Inflammation	Human/Mouse	cagA+ and vacA+
ATCC43504 (64)	Inflammation	Human/Mouse	cagA+ and vacA+
ATCC 51932 (64)	Mild disease	Human/Mouse	cagA- and vacA+
NCTC11637 (65)	Cellular apoptosis	Human/Gerbil	cagA+ and vacA+
7.13 (66)	Duodenal ulcer	Human/Gerbil	cagA+ and vacA +
26695 (63)	Mild inflammation/ulcer	Human/Piglet	cagA+ and vacA +
TN2GF4 (67)	Intestinal metaplasia	Human/Gerbil	cagA+ and vacA +
42GX (67)	Gastritis	Human/Gerbil	cagA+ and vacA +

Current vaccine candidates target an array of *H. pylori* antigens can enhance host immunity, despite the delivery method (oral, intranasal mucosal, subcutaneous injection) (68–70), and vaccine composition (primarily peptide vaccines and RNA vaccines) (71, 72). However, they have not yet achieved consistent, long-term protection against *H. pylori* infection. The immune response against *H. pylori* primarily orchestrated in the gastric mucosa, where reminiscent of intestinal Peyer’s patches play a crucial role (73). he recognition of bacterial virulence factors or pathogen-associated molecular patterns (PAMPs) by pattern recognition receptors, such as TLR4 or Nod1, triggers the activation of the MyD88-NF-κB pathway and other signaling cascades. This cascade leads to the release of inflammatory cytokines and the initiation of adaptive immune responses, particularly the CD4+ T-cell response.

T-cell assistance facilitates the activation and differentiation of follicular B-cells into plasma cells, which secrete IgA. Secretory IgA is pivotal in mucosal immunity, neutralizing various toxins, while IgG appears to play a less significant role in this context (74). The development of *H. pylori* vaccines have predominantly focused on subunit oral vaccines, designed to efficiently activate mucosal responses. These vaccines, containing single and highly specific peptides devoid of toxic components, theoretically should not elicit long-term intense inflammatory reactions.

Extensive research has demonstrated that oral subunit vaccines generally elicit Th1/Th17 responses and stimulate the production of secretory IgA upon entering the body, significantly enhancing pathogen clearance. However, a clinical trial in China revealed a gradual decline in the protective efficacy of oral vaccines over time, accompanied by decreasing antibody levels (75). Consequently, current research efforts are directed towards enhancing the sustained protective capacity of vaccines, which hinges on the immunogenicity of the vaccine peptides and the selection of appropriate adjuvants.

A recent breakthrough study demonstrated that an LNP-mRNA vaccine for influenza virus maintained superior lung mucosal immune responses compared to protein vaccines, facilitating the formation of germinal center follicular B-cells and memory T-cells (76). This research also highlighted the potential of LNP as a potent immunostimulatory adjuvant, not only for mRNA vaccines but also for enhancing the efficacy of various vaccine types. These findings open new avenues for vaccine development against *H. pylori* and other mucosal pathogens, potentially revolutionizing our approach to preventive medicine.

Recent advances in vaccine technology offer new avenues for exploration. A multi-epitope oral vaccine, incorporating four *H. pylori* virulence factors (Urease, NAP, HSP60, and HpaA), showed promising results in murine models, eliciting robust systemic and mucosal immune responses (77). This approach highlights the potential of tailoring comprehensive and multi-target vaccine strategies to overcome the challenges posed by H. pylori’s genetic diversity, a key component of precision therapy approaches.

The virulence of *H. pylori* strains is intricately linked to genetic factors, particularly the cagA and vacA genes. CagA-positive strains correlate with increased risk of peptic ulcers and gastric cancer, while vacA gene variations (s1/m1, s1/m2, s2/m2) modulate cytotoxicity and disease outcomes (78–82). These findings underscore the importance of tailoring vaccine strategies to address the molecular heterogeneity of *H. pylori* strains.

Nucleic acid vaccines represent a cutting-edge approach, offering the potential to induce robust humoral and cellular immunity against conserved epitopes across multiple *H. pylori* subtypes (83–85). This technology circumvents challenges associated with oral vaccine delivery, such as gastric acidity and mucosal immune induction, presenting a promising avenue for future research.

## 8 Current clinical trials and challenges in *H. pylori* vaccine development

The urgent need to combat *H. pylori* infection has driven the development of various vaccine candidates (**Table 2**). Despite extensive efforts, significant success remains elusive. Clinical studies primarily assess vaccine efficacy through measurements of specific antibody levels and post-vaccination adverse reactions. However, a critical gap exists in evaluating the vaccines’ ability to prevent *H. pylori* infection, limiting our understanding of their real-world effectiveness (70). A notable Phase I/II clinical trial conducted by Peter et al. investigated an oral vaccine containing three recombinant *H. pylori* antigens: VacA, CagA, and neutrophil-activating protein (NAP). While vaccinated volunteers exhibited significantly higher levels of *H. pylori*-specific antibodies compared to the control group, the vaccine failed to demonstrate superior infection prevention. Surprisingly, over half of the control group experienced spontaneous infection clearance, highlighting the complexity of *H. pylori* immunology and the challenges in vaccine development.

**Table 2:** Clinical trials of *H. pylori* vaccines.

Clinical Trials ID	Antigen(s)	Last Update	Clinical Stage	Route	Results
——	rUrease	1999 (88)	Phase I	Oral	No protection
——	Formalin-inactivated *H. pylori* whole-cell vaccine	2001 (68)	Phase I	Oral	No protection.
——	rUrease	2002 (89)	Phase I	Oral	Low seroconversion; diarrhea.
——	UreA, UreB	2004 (90)	Phase I	Oral	Partially increase immunity.
——	UreA, UreB	2008 (91)	Phase I	Oral	No protection
NCT00613665	NAP+CagA+VacA	2013 (Not yet published)	Phase I	intramuscular	Not provided
NCT02302170	UreB	2015 (75)	Phase III	Oral	Increase of serum IgG and salivary IgA. partial protection
NCT00736476	VacA+CagA+NAP	2018 (70)	Phase I/II	Intramuscular	Increasing mean concentrations of antibodies specific to CagA. No protection.
NCT03270800	IMX101: gGT, HpaA, CTA1-DD	2019 (Not yet published)	Phase I/II	Intradermal/sublingual.	Low immunogenicity.

rUrease, recombinant urease; Ure, urease; CagA, cytotoxin-associated gene A; VacA, vacuolating cytotoxin A; NAP, neutrophil-activating protein; gGT, gamma-glutamyl transpeptidase; HpaA, *H. pylori* adhesin A; CTA1-DD, cholera toxin A1 subunit fused to two IgG binding D motives from Staphylococcus aureus protein A.

The limited efficacy of current vaccines can be attributed to several factors. *H. pylori* exhibits considerable genetic diversity, particularly in its virulence factors, which display high antigenic variability. Antigens with low conservation between different *H. pylori* strains are prone to evading immune recognition (86). Moreover, virulence factors targeted by vaccines may be lost following vaccination, rendering the immune response ineffective. The high genetic variability of *H. pylori* enables rapid adaptation to the selective pressure imposed by vaccination, posing a significant obstacle to vaccine efficacy. Enhancing vaccine immunogenicity while mitigating immune escape mechanisms remains a central challenge in developing effective immunization strategies against *H. pylori* (87).

The development of universal vaccines with multi-epitope tailored to multi-pathogenic *H. pylori* subtypes holds significant promise. However, realizing this potential requires overcoming the complexities of antigen variability and immunogenicity assessment. Leveraging advanced bioinformatics and machine learning or Artificial Intelligence algorithms may accelerate the design and optimization of next-generation *H. pylori* vaccines, potentially revolutionizing our approach to gastric cancer prevention through precision medicine and targeted therapy strategies.

## 9 AI breakthroughs redefine neoantigen-based anti-bacterial vaccines development

### 9.1 Computational tools in vaccine design

AI has revolutionized vaccine development, enhanced efficacy and accelerating timelines. By targeting microscopic molecules, modern vaccines offer advantages over traditional approaches, with AI methods playing a crucial role in their design (92). These methods reduced the number of proteins to be studied and was able to recognize small amounts of antigens present, enhance stability, streamlining the development process and offering new insights for precision medicine (93).

Reverse vaccinology (RV), introduced by Rino Rappuoli in 2001, leverages genomic data to identify potential vaccine targets (94). This approach has spawned numerous computational tools, including PanRV (95), VaxiJen (96), Vaxign (97), Antigenic (98) etc. NERVE, the pioneering bacterial vaccine prediction tool based on RV principles, considers factors such as antigen foreignness, adhesion status, and localization to improve vaccine safety and efficacy (99). Machine learning has further advanced vaccine development. Vaxign-ML, utilizing extreme gradient boosting, incorporates 509 features and was trained on a comprehensive dataset of bacterial protective antigens (100). Following this review provides a list of these methods from 2006 to 2021 and their key characteristics (**Table 3**). This approach exemplifies the potential of AI in vaccine design, offering rapid and accurate predictions of vaccine candidates.

**Table 3:** The list of AI prediction tools based on RV and their characteristics.

Name	Method	Crucial Features
NERVE (2006)	Expert System	localization, adhesin probability, foreignness.
VaxiJen (2007)	DA-PLS	Protein Sequence.
Vaxign (2008)	Feature List	localization, transmembrane helices, adhesin probability, sequence conservation, foreignness.
Jenner-predict (2013)	Feature List	localization, transmembrane helices, Immunogenicity, functional domain.
VacSol (2017)	Feature Selection Pipeline	Subcellular localization, Transmembrane helices, B-cell and T-cell epitopes, Non-host homologous proteins, Virulence factors.
Antigenic (2019)	Random Forest, SVM-RFE	Amino acid composition, Dipeptides, Tripeptides, n-Gapped Dipeptides, n-Grams.
PanRV (2019)	Feature Selection Pipeline	Pangenome Estimation Module, Reverse Vaccinology Module Functional Annotation Module, Antibiotic Resistance Association Module.
Vaxign-ML (2020)	XGBoost	localization, Adhesin probability, Transmembrane helix, physchemical properties (509 features).
Vax-ELAN (2021)	Feature Selection Pipeline	Subcellular localization, Secretory/non-secretory, Stability, Cleavage sites, Adhesion properties, MHC binding, Transmembrane helices, Essentiality, Virulence, Molecular weight, Non-homology with host proteins.
NUCC (2024)	CNN, FCNN	Protein sequence, HLA Typing, MixMMHCpred result, NetMHCpanresult, NetMHCstabpan result

Despite promising advancements, challenges remain. The limited availability of training data and the complexity of immunogenicity factors, including MHC affinity and TCR avidity, necessitate further refinement of these tools (101). However, these tools often lack consideration for these finer details during their design. For bacterial immunity, MHC class II affinity prediction is particularly crucial yet complex (102–104). NetMHCIIpan-4.0 represents a significant advancement in MHC class II binding affinity prediction. It is based on NNAlign MA machine learning framework, adopting deconvolution method and pseudo-labeling strategy (105). In detail, NetMHCIIpan-4.0 leverages binding affinity (BA) data from single MHC allele sequences to perform deconvolution, identifying anchor positions and amino acid preferences, which define binding motifs (105). Subsequently, it generates single-allele specific pseudo-labels from spectrometry-eluted ligand (EL) data (multi-allele MHC binding). The modeling method allowed NetMHCIIpan-4.0 to be trained on larger datasets, learning more comprehensive information (105). Moreover, compared to single-allele prediction tools, NetMHCIIpan-4.0 has been trained on data covering a total of 116 different MHC II molecules, which significantly enhances its overall generalization capability and allows for accurate MHC II affinity predictions (105). Recently, most tools have applied deep learning models, such as Convolutional Neural Networks, Transformers (106), and BERT (107), to project protein sequences into high-dimensional spaces, promise even more accurate predictions of MHC class II binding motifs. The list of tools predicting MHC class II affinity was as follow (**Table 4**).

**Table 4:** The list of AI tools for predicting MHC class II affinity and their characteristics.

Name	Method	Database	crucial features
SMM-align	PSSM	IEDB	optimized Blosum50, peptide flanking residues
NetMHCIIpan-4.0	NNAlign_MA	BA data, EL data	Protein sequence
BERTMHC	protein BERT	IEDB, EL data	Protein sequence

As these computational methods evolve, incorporating increasingly complex biological factors, they pave the way for a new generation of vaccines with enhanced safety, specificity, and immunogenicity. This convergence of biology, computer science and precision medicine herald a new era in vaccine development, offering hope for rapid responses to emerging pathogens and improved global health outcomes through both vaccines and targeted therapies.

### 9.2 AI and bioinformatics applications in vaccine candidate discovery

To date, thousands of vaccine candidates have been developed using advanced technologies to target a wide range of lethal pathogens (108). In recent years, artificial intelligence (AI) and bioinformatics have emerged as pivotal tools in vaccine design, particularly for COVID-19. The integration of these technologies facilitated the rapid development of COVID-19 vaccines in under a year, significantly contributing to epidemic prevention and control—an efficiency unattainable through traditional methods (93).

Modern vaccine design now comprehensively creates safe, stable, and effective vaccines by determining epitopes, utilizing epitope immunoinformatics, and assessing vaccine epitope structure and stability. Reverse vaccinology (RV) is employed to identify genes with potential epitopes, with tools such as Vaxign ML being widely used (100, 109). BLASTp comparisons of epitope sequences against human genes help exclude sequences with homology to human epitopes, mitigating the risk of autoimmune reactions post-vaccination (110).

To identify suitable epitope antigens, it is crucial to utilize appropriate epitope immunoinformatics analysis tools to evaluate the potential of epitopes to elicit robust immune responses. Tools like NetMHCIIpan-4.0 and Epitopemap (111), aid in understanding the genetic polymorphisms of major histocompatibility complex (MHC) classes I and II in the target population, as well as predicting cytotoxic and helper T lymphocyte epitopes. Some studies employ I-TASSER (112) to elucidate the conformational characteristics of epitopes and to identify antigenic epitopes with enhanced immunogenicity.

The insights gained from these vaccine development experiences serve as valuable references for the development of *H. pylori* vaccines, potentially accelerating the process and improving efficacy.

### 9.3 Limitations of AI-designed vaccines

AI and bioinformatics-based vaccine design faces several key limitations. Firstly, epitope antigens are typically confined to protein-encoding genes, whereas traditional approaches can target a broader range of biological entities, including polysaccharides (113). Secondly, certain epitope antigens in viral proteins may evade human immune recognition, failing to elicit an effective immune response. Thirdly, while AI-designed vaccines are often evaluated based on B and T cell immune responses, such as specific antigen antibody levels, elevated antibodies do not always translate to strong preventive or therapeutic effects, as demonstrated by Peter et al. (70). Lastly, validating the efficacy of vaccines for certain pathogens is challenging due to the difficulty in selecting appropriate animal models (93).

Despite AI’s potential to expedite *H. pylori* vaccine development, its application faces hurdles. AI model training requires extensive, high-quality data, but biological experiments are typically low-throughput and time-intensive. For instance, the T cell activation experiments in the IEDB are insufficient for robust AI model training (114). High-throughput experiments, such as IP-MS studies on MHC II (including HLA-DR, HLA-DQ, and HLA-DP) under various conditions, can significantly expand training sets, potentially surpassing one million data points for MHC II affinity prediction (105). Integrating high and low-throughput biological experimental data could further enhance AI model performance.

Bias presents another significant challenge in AI model training. The tendency of researchers to prioritize positive results over negative ones leads to discrepancies between training and true distributions. This issue can be addressed through pseudo-label training, enabling AI models to learn within the true distribution (105).

Furthermore, the prevalence of weak functional molecules results in a severe imbalance between negative and positive results in unlabeled samples (115). This imbalance compromises the representativeness of negative data and hinders effective learning of positive data patterns when large amounts of negative data are introduced, ultimately impeding AI model training. Common strategies to address this include resampling to adjust the ratio of training data or applying weighted corrections.

## 10 Perspectives: precision therapy and AI-driven vaccine design

The persistent challenge of *H. pylori* infection, a Class I carcinogen, contributes to significantly global gastrointestinal diseases. Despite extensive research efforts, conventional therapeutic approaches have been hampered by antibiotic resistance and recurrence, underscoring the urgent need for innovative strategies. This review illuminates the potential of next-generation *H. pylori* vaccines in bolstering host immunity and mitigating infection-associated gastric cancer risk (1).

While recent advancements in understanding immune mechanisms and inflammatory responses have provided valuable insights, their translation into effective preventive vaccines remains elusive. Although some clinical trials have shown promise, significant hurdles persist, including suboptimal efficacy and adverse effect. We propose a paradigm-shifting approach rooted in precision medicine: the development of tailored, multiepitope antigens targeting diverse *H. pylori* pathogenic subtypes. By leveraging cutting-edge artificial intelligence tools, we envision the creation of highly specific *H. pylori* neoantigen vaccines for gastric cancer prevention. This innovative strategy not only promises to address the limitations of current triple-antibiotics therapies, but also has the potential to revolutionize our approach to oncogenic pathogens more broadly. The integration of AI-driven vaccine design with precision therapy concepts opens new avenues for personalized interventions, potentially offering a transformative solution to the global burden of *H. pylori*-associated diseases.

As we stand at the cusp of this exciting frontier, the convergence of AI, immunology, and precision medicine holds immense promise for reshaping the landscape of infectious disease management and cancer prevention. This approach may serve as a blueprint for tackling other persistent pathogen-associated carcinogenesis, heralding a new era of tailored therapeutic strategies with far-reaching implications for global health.
